# Multimodal Imaging and Hybrid Scanners

**DOI:** 10.1155/2007/45353

**Published:** 2007-06-03

**Authors:** Haim Azhari, Robert R. Edelman, David Townsend

**Affiliations:** ^1^Department of Biomedical Engineering, Technion – Israel Institute of Technology, Haifa 32000, Israel; ^2^Department of Radiology, Evanston Northwestern Healthcare, 2650 Ridge Avenue, Evanston, IL 60201, USA; ^3^Department of Medicine and Radiology, University of Tennessee Medical Center, 1924 Alcoa Highway, Knoxville, TN 37920, USA

There is an old tale of three blind men who were brought to the zoo for the first time and allowed to touch the elephant. On their way home, they shared this exciting experience. “An elephant is a long, flexible, and cylindrical creature,” said the man who had touched the elephant's trunk. “No! It is a thin and flat creature,” said the man who had touched the elephant's ear. “No, no! An elephant is rough and rigid like a tree stem,” disagreed the third person who had touched the elephant's leg. And the truth is that all three men were right!

When trying to see the invisible with our medical imaging systems, we are much like those three blind men. With each imaging modality, we shed some light on a different aspect of the general physiological picture. Although in some cases one modality may suffice to provide a definitive clinical answer, this is not the case in many other situations. Multimodal imaging (MMI) is needed for three basic reasons: (a) to acquire *complementary* information which may be needed to reach a definitive diagnostic conclusion, exclude certain pathologies, or obtain quantitative values (e.g., [1, 2]); (b) to create *synergism* by data fusion (i.e., to provide added information and new images which are more informative than the individual source images); (c) to plan *therapeutic* procedures and monitor treatment (e.g., [3, 4]).

An ideal MMI system or method should be capable of performing all three tasks mentioned above. Naturally, that requirement might be too demanding in terms of technological capabilities and operational considerations. Hence, diagnostic and therapeutic systems are commonly separated. However, one may see in the near future more system integration in the form of image-guided therapy.

There are several technical issues that are associated with MMI. A prerequisite is to obtain effective fusion and display of the data (e.g., [5–7]). Accurate spatial (and maybe also temporal) alignment is crucial for effective data fusion. There are basically two approaches for achieving coregistration. The first, which may be called the “hardware” approach, utilizes a hybrid design comprising two (or more) imaging modalities that are contained within a single device. The advantage of this approach is that the imaging modalities acquire data sequentially while the patient lies on the bed. The disadvantage is the need for dedicated MMI equipment which may be cumbersome or costly.

The second approach for achieving coregistration is the “software-” based approach. With this approach, image properties and tissue geometry and texture are used as clues for aligning the data sets. Alignment is thus achieved by manipulating the acquired data under certain optimization constraints or 3D model to achieve the best (most probable) match (e.g., [8–11]). Of course this approach is susceptible to noise and artifacts, but on the other hand it allows better versatility, and in many cases may be applied successfully to scans performed on different occasions and at different locations. Nevertheless, it is now widely recognized that the merger of information is more efficiently achieved by the hardware approach. The recent (2001) introduction of hybrid scanners has led to an expansion of this approach through the rapid adoption of the technology into the clinical arena.

One of the most promising examples of MMI hybrid systems that is currently demonstrating a significant clinical impact is the combination of CT with nuclear imaging, and specifically positron emission tomography (PET). Following the development of a prototype in the late 1990s [12], the first commercial combined PET/CT scanner was introduced in 2001 and since then, close to 2000 of such devices from different vendors have been installed in clinics worldwide. Both CT and PET technologies continue to advance and since 2006, new PET scanners are now only available in combination with CT. The MMI technology available clinically has demonstrated particular impact in staging malignant disease [13, 14] and in monitoring response of the disease to therapy. The recent incorporation of high-speed, multislice CT scanners with PET also opens up the potential for applying this technology to cardiac disease.

Another attractive modality for MMI is MRI. Although MRI imposes severe restrictions on the imaging environment, it offers a broad spectrum of scan types and image contrast. Compared with CT, MRI offers greater soft-tissue contrast, better capability for quantitation of function (e.g., measurement of blood flow or tissue metabolism), and potentially new types of molecularly targeted contrast agents. Efforts for combining MRI with other modalities (e.g., PET/MRI and ultrasound/MRI) are currently under development.

Another aspect of MMI is the development of multimodal contrast enhancing materials. Such materials can be used in the form of a “fit-all” type of marker (e.g., [15]). Thus, their signals can be used as control points for 3D alignment. Alternatively, they can be used as standard contrast agents used for disease detection and characterization (e.g., [16]).

In conclusion, considering the current tends in radiology, it can be expected that MMI devices will become increasingly available in the clinical arena. PET/CT has already made an important clinical contribution to patient care for oncology, while the new combined SPECT/CT designs are enhancing SPECT applications and improving physicians' confidence with image interpretation. No doubt, new combinations of hybrid devices will appear in the clinical arena and in many situations. As demonstrated by PET/CT in the oncology field, they will become the primary imaging option. A PET/MR design for simultaneous acquisition of PET and MR has recently acquired the first patient images, and a combined PET and ultrasound device is also under development for breast imaging. For many reasons, therefore, hybrid imaging devices are finding widespread acceptance within the clinical environment and some are already contributing to patient care and management. There is little doubt that this trend will continue in the future with an increasing reliance on MMI devices for medical imaging, thereby ensuring that all involved can be satisfied that they will eventually obtain a true and consistent picture of the elephant.

*Haim Azhari* *Haim Azhari*

*Robert R. Edelman* *Robert R. Edelman*

*David Townsend* *David Townsend*
